# The Pharmacometabodynamics of Gefitinib after Intravenous Administration to Mice: A Preliminary UPLC–IM–MS Study

**DOI:** 10.3390/metabo11060379

**Published:** 2021-06-11

**Authors:** Billy Molloy, Lauren Mullin, Adam King, Lee A. Gethings, Robert S. Plumb, Ian D. Wilson

**Affiliations:** 1 Waters Corporation, Stamford Rd, Wilmslow SK9 4AX, UK; Billy_Molloy@waters.com (B.M.); mullin.lauren@yahoo.com (L.M.); adam_king@waters.com (A.K.); Lee_Gethings@waters.com (L.A.G.); 2 Waters Corporation, Milford, MA 01757, USA; Rob_Plumb@waters.com; 3 Division of Systems Medicine, Department of Metabolism Department of Metabolism, Digestion and Reproduction, Imperial College London, London SW7 2AZ, UK

**Keywords:** gefitinib metabolomics, pharmacometabonomics, pharmacometabodynamics, rapid profiling, metabolite identification

## Abstract

The effects of intravenous gefitinib (10 mg/kg), an anilinoquinazoline thymidylate kinase inhibitor (TKI), selective for the epidermal growth factor receptor (EGFR), on the urinary metabotypes of mice were studied. We hypothesized that, in response to the administration of gefitinib, there might be significant changes in the excretion of many endogenous metabolites in the urine, which could be correlated with the plasma pharmacokinetics (PK) of the drug. In order to investigate this conjecture, urine from male C57 BL6 mice was collected before IV dosing (10 mg/kg) and at 0–3, 3–8, and 8–24 h post-dose. The samples were profiled by UPLC/IM/MS and compared with the profiles obtained from undosed control mice with the data analyzed using multivariate statistical analysis (MVA). This process identified changes in endogenous metabolites over time and these were compared with drug and drug metabolite PK and excretion. While the MVA of these UPLC/IM/MS data did indeed reveal time-related changes for endogenous metabolites that appeared to be linked to drug administration, this analysis did not highlight the presence of either the drug or its metabolites in urine. Endogenous metabolites affected by gefitinib administration were identified by comparison of mass spectral, retention time and ion mobility-derived collision cross section data (compared to authentic standards wherever possible). The changes in endogenous metabolites resulting from gefitinib administration showed both increases (e.g., tryptophan, taurocholic acid, and the dipeptide lysyl-arginine) and decreases (e.g., deoxyguanosine, 8-hydroxydeoxyguanosine, and asparaginyl-histidine) relative to the control animals. By 8–24 h, the post-dose concentrations of most metabolites had returned to near control values. From these studies, we conclude that changes in the amounts of endogenous metabolites excreted in the urine mirrored, to some extent, the plasma pharmacokinetics of the drug. This phenomenon is similar to pharmacodynamics, where the pharmacological effects are related to the drug concentrations, and by analogy, we have termed this effect “pharmacometabodynamics”.

## 1. Introduction

Metabolic phenotyping (metabonomics/metabolomics) has previously been shown to have utility in predicting likely drug response based on pre-dose metabolite profiles. This property of an organism’s metabotype was first demonstrated by Clayton et al. for acetaminophen (paracetamol) in both rats [1] and humans [2]. This phenomenon, originally termed phamacometabonomics by its’ discovers (reviewed in e.g., [3,4]), and subsequently as pharmacometabolomics by others [5], has stimulated much research in this area [3,4,5]. The ability to predict a response, or a lack thereof, based on pre-dose metabotypes has led to the advocacy of the use of pharmacometabonomic/pharmacometabolomic approaches in personalized medicine. In addition, metabolic profiling has obvious applications in examining the effects of drugs and toxins to seek mechanistic insights into modes of action.

Similarly, given the general nature of metabolic phenotyping, it is also clearly possible to use untargeted metabolic profiling to look for the “off target” pharmacological effects of drugs. Studying the global effects of drugs in this way may, in addition to supporting mode of action investigations and helping to understand adverse effects, also suggest alternative uses for them, and such drug repurposing represents a very active area of research [6]. One obvious area for development is not simply to link pre-dose profiles with likely efficacy, or even the effects of the drug on the metabolome following dosing, but to link the pharmacokinetics of the drug and its metabolites with the time-related changes in the metabolic phenotype of those to whom it has been administered. This is clearly similar in concept to pharmacodynamics and, to distinguish it from “conventional” pharmacometabonomics, a term such as “pharmacometabodynamics” might be appropriate.

Here, we report some preliminary results on the effects on the urinary metabolic profiles of mice following the IV administration of the anticancer drug gefitinib (Iressa^®^), an anilinoquinazoline thymidylate kinase inhibitor (TKI) (structure in Figure S1). Gefitinib, which is selective for the epidermal growth factor receptor (EGFR), was developed as an oral cancer treatment directed against non-small cell lung cancer (NSCLC), and is effective in patients with specific mutations of EGFR [7,8,9].

Gefitinib has been shown to be well absorbed with a good bioavailability, but it is subject to extensive biotransformation in both preclinical species [10,11,12,13,14,15] and humans (e.g., [11,15,16,17,18,19]) to a large number of metabolites. As a result of these in vivo studies, and a number of in vitro [20,21,22,23] investigations, it is known that gefitinib metabolism involves a wide combination of biotransformations. These include O-demethylation, oxidative metabolism of the morpholine ring, and oxidative defluorination (e.g., [11,20,21,22,23]), much of which is mediated via CYP3A4 and 3A5, as well as contributions from CYP2D6 [21,22]. More recently, the further biotransformation of some of these oxidative metabolites to sulfate and glucuronide conjugates has been observed [14,15,16,19].

While, as will be clear from the above, the pharmacokinetics (PK) and metabolic fate of the drug have been well studied, the consequences of gefitinib administration to the metabolome have not. However, as has long been known in toxicity studies where metabolic phenotyping has been performed, there are often significant, time-dependent changes in the profiles of endogenous metabolites in response to the administration of a toxin. So, in an acute toxicity study, early changes in the metabolic phenotype can indicate the onset of metabolic dysregulation and the development of tissue damage [24,25]. If the insult is such that the organism is able to recover and repair the damage caused by the toxin, metabolite profiles will begin to normalize as repair and homeostasis occurs. Similarly, as discussed above, the administration of a therapeutic drug may also be associated with changes that will be regulated by the pharmacokinetics of its actions, and this can also be expected to result in time-dependent changes in the metabolome.

Here, similarly to pharmacodynamic studies, we investigated the effects of gefitinib dosed intravenously to mice on the urinary profiles of endogenous metabolites in order to see whether there were pharmacometabodynamic effects of the drug on the metabolomes of these animals. This was undertaken using both UHPLC/MS and UHPLC/IM/HRMS, following the intravenous administration of the drug at 10 mg/kg. We also examined the potential of metabolic phenotyping combined with unbiased multivariate statistical analysis (MVA) to highlight the presence of drugs and metabolites in the urine as part of an unbiased approach to drug metabolite detection.

## 2. Results and Discussion

Since the earliest days of modern untargeted metabolic phenotyping, the potential for providing a means of rapidly and efficiently detecting both xenobiotic and endogenous metabolites in biological fluids has been apparent, e.g., see [26,27,28,29]. However, these early studies were often associated with relatively high dose drugs (e.g., paracetamol (acetaminophen) [26,27] or oxpentifylline [28]) or toxicological investigations, where high doses were employed to achieve a toxic effect [29]. Developments in chromatographic analysis and mass spectrometry, together with the application of IM spectrometry, have provided new and efficient methods to investigate the effect of drugs on the endogenous metabolome, and to correlate these with drug/metabolite exposure (e.g., see [30,31,32,33,34]).

In particular, the increasing sensitivity of LC/MS offers the opportunity of using microsampling to investigate the pharmacokinetics and metabolic fate of drug candidates in early drug discovery very efficiently. However, in addition, these advances also enable the samples gathered in DMPK studies to be used to investigate drug effects on the metabolome at low, essentially pharmacological, doses, rather than those associated with toxicity. This strategy was employed here with conventional DMPK profiling performed initially [14], followed by the metabolic phenotyping of the same samples, as described below.

### 2.1. Plasma Pharmacokinetics of Gefitinib and Metabolites

In the initial phase of our study on gefitinib in mice, the pharmacokinetics of the drug and some of its metabolites were determined [14]. This analysis showed that IV administration at 10 mg/kg was associated with a mean maximum observed plasma concentrations of gefitinib of 4.4 µg/mL. These plasma concentrations were detected 6 min post-dose (the first time point measured) and the concentrations of the drug then declined with a half-life (T_1/2_) of 2.6 h. Gefitinib was no longer detectable in the samples collected 24 h post-dose (see Figure S1).

Metabolites of the drug were also detected at the earliest time points studied, with high concentrations for the morpholino carbonyl compound (M605211) and the O-desmethyl metabolite (M523595) at 6 min post-dose, with maximum observed concentrations noted 1 h post-dose (see Figure S1). The concentrations of these metabolites then declined in parallel with those of gefitinib, and were also undetectable in the 24 h samples [14]. These results showed, in line with previous studies in rodents [10,11,12,13,14,15], that the drug was rapidly metabolized, producing a large number of metabolites, which were detected in both the circulation and urine [14].

### 2.2. Untargeted Analysis of Urine Including Gefitinib and Metabolites

In determining the metabolism of gefitinib in these mice [14], we performed “conventional” metabolic profiling to detect and characterize the metabolites of the drug excreted in the urine. The same analyses also provided the data necessary to look at the changes in the profile of endogenous metabolites. The urine samples analyzed were obtained from mice housed in groups of five animals/metabowl, and thus represent pooled collections from each of these groups.

**Figure 1:**
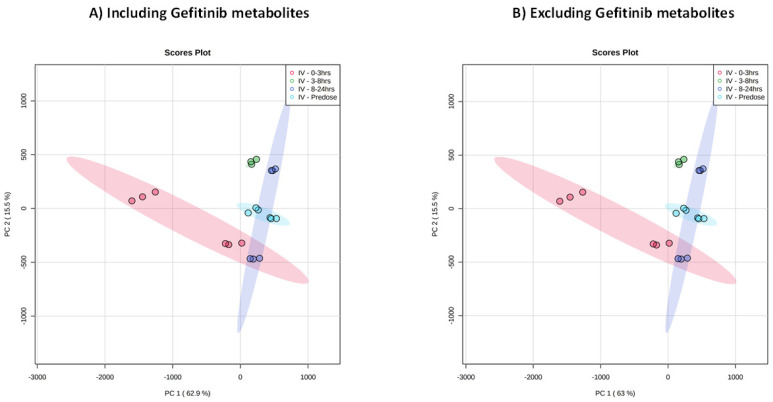
PCA of positive ESI LC–IM–MS data for urine obtained from male C57Bl6 mice dosed intravenously with 10 mg/kg gefitinib for the periods of pre-dose (light blue) and 0–3 (red), 3–8 (green), and 8–24 h (dark blue). These data were obtained following analysis (each urine pool in triplicate) with (**A**) MS data for gefitinib and metabolites included and (**B**) with MS data for gefitinib and metabolites removed, demonstrating the lack of any contribution to the separation as a result of drug-related material in the urine.

Given the obvious need to exclude gefitinib and its metabolites from the untargeted endogenous metabolic profiling data, we also took the opportunity to investigate the approach (originally shown for LC/MS by Plumb et al. [30]) of using MVA to detect drug-related features in an unbiased way. When the untargeted LC/MS data obtained for each of the time period collections (pre-dose, and 0–3, 3–8, and 8–24 h post-dose) were analyzed in this way, differences were readily observed between control and IV dosed samples by PCA (Figure 1 for positive ESI and Figure S2 for negative ESI). However, despite the clear detection of gefitinib and its metabolites in the urine obtained previously [14], and unlike the situation reported for a number of other compounds, e.g., [30,31,32,33,34], none of the ions associated with them contributed to the observed PCA separation. Thus, as shown in Figure 1A and B for the positive ion data, the removal of all of the previously identified ions associated with gefitinib and its metabolites [14] had no effect on the observed PCA score plot. A similar result was also seen for the negative ion data (not shown), and no signals for either the drug or its metabolites were found to be significant through PCA.

**Figure 2:**
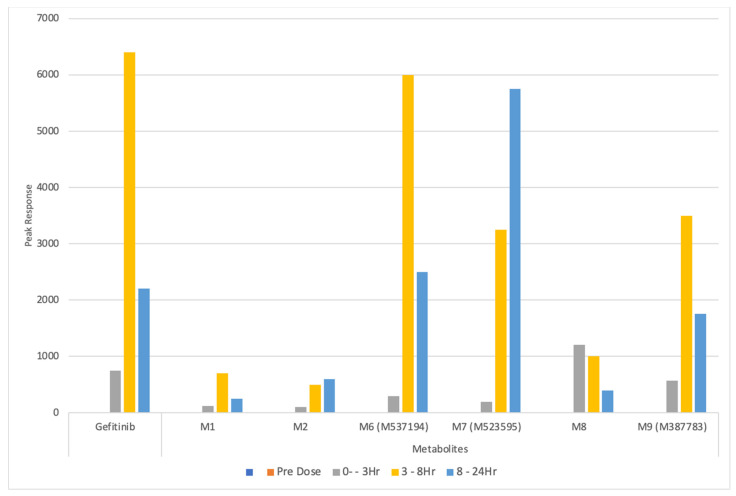
Relative amounts (based on peak response) for gefitinib and selected metabolites in the urine of IV dosed mice for the time points obtained from pooled pre-dose (orange) and 0–3 h (grey), 3–8 (yellow), and 8–24 h (blue) post-dose samples [14]. As indicated, no signals for gefitinib or its metabolites were detected in the pre-dose samples, while the relative rates of excretion of the drug and its metabolites showed compound-dependent profiles. The pharmacokinetic (PK) plasma profiles reported in [14] for gefitinib, M605211, and the O-desmethyl metabolite M7 (M523595) are provided in Figure S1.

This result is somewhat disappointing, as the dose administered at 10 mg/kg, is not insignificant, and of the order frequently encountered in drug discovery settings. In addition, as noted above, when these UPLC/IM/MS data for urine were examined using “conventional” methods, the drug and its metabolites were readily detected [14]. This in vivo result contrasts with the findings of in vitro incubations of gefitinib reported by Liu et al. [23]. This study employed human and mouse liver microsomes, as well as recombinant CYP450s, to determine the nature of the reactive metabolites produced by the oxidative metabolism of gefitinb. Upon incubation of the drug at concentrations of 30 µM, a large number of oxidative metabolites, including a range of reactive metabolites, were detected by MVA, using the supervised approach of OPLS-DA [23]. However, in vivo radiolabeled studies have shown that the bulk of the dose of gefitinib given to preclinical species, such as rats and dogs, was excreted in the feces [11] with under ca. 10% eliminated via the urine. The qualitative urinary excretion data for gefitinib and a number of its metabolites obtained in this mouse study [14] are illustrated in Figure 2, with the plasma pharmacokinetic data for gefitinib and the major circulating O-demethylated and morpholino-carbonyl metabolites (M523595 and M605211) shown in Figure S1. As this figure shows, these compounds declined rapidly from their peak observed concentrations and, as noted above, were no longer detected in circulation 24 h post-dose (see Figure S1).

If we examine the urinary excretion profile of gefitinib and its metabolites, it is clear that excretion was rapid, as they were present even in the 0–3 h post-dose urine. Indeed, concentrations of the morpholine ring-opened O-desmethyl metabolite (M8) peaked in the 0–3 h collection. For gefitinib itself, and e.g., the morpholine-ring-M6 (M537194) and desfluorophenol-M9 (M387783) metabolites, respectively, the maximum amounts detected in the urine were present in the 3–8 h post-dose samples, reducing in the 8–24 h urines.

There was also a third group of metabolites where, although detectable in both the 0–3 and 3–8 h collections, the maximum amounts observed were in the 8–24 h urine, typified by the O-desmethyl metabolite M7 (M523595). While readily detected using conventional methods, the absence of these compounds as discriminating features in the PCA analysis is more disappointing when compared with some earlier work, e.g., [23,30,31,32,33,34]. However, given that the removal of gefitinib-related features from the PCA had no discernible effect on the score plots for PCs 1 and 2, it is unsurprising that the examination of the data confirmed that none of the top 100 discriminating ions were gefitinib-related. This probably reflects the combination of the major route of excretion being via the feces and the low dose of gefitinib administered. Both factors should be compared with the much higher amounts (often several 100 mg/kg) administered in toxicity studies on, e.g., paracetamol (acetaminophen) [31,33] or 2-bromophenol [33,34], where significant urinary excretion of drug/xenobiotic-related metabolites occurred and contributed to the PCA. Thus, the extensive metabolism of gefitinib, to a large number of metabolites, added to the complexity of the sample, but clearly did not contribute significant features to the profile. Some support for this view that high doses are beneficial for a metabolomic approach to drug metabolism studies on this class of compound comes from a recent study on the TKI inhibitor nintedanib (used for the treatment of idiopathic pulmonary fibrosis (IPF) and, when combined with docetaxel as a treatment for NSCLC) [35]. Here, a dose of 200 mg/kg to mice was employed, which allowed 19 metabolites to be detected and characterized in the urine and feces following data analysis using OPLS-DA. The same authors were able to characterize 38 metabolites of the drug agomelatine (a melatonin analogue) in human liver microsomal incubations and the excreta of mice dosed orally at 50 mg/kg (again using OPLS-DA to highlight ions of interest for characterization) [36]. However, despite the failure to find gefitinib and its metabolites in urine using untargeted metabolite profiling combined with MVA, it is interesting to observe how the endogenous metabolite profiles varied considerably with systemic exposure to gefitinib (described in [14] and illustrated in Figure S1**)**.

### 2.3. Untargeted Metabolic Phenotyping

**Figure 3:**
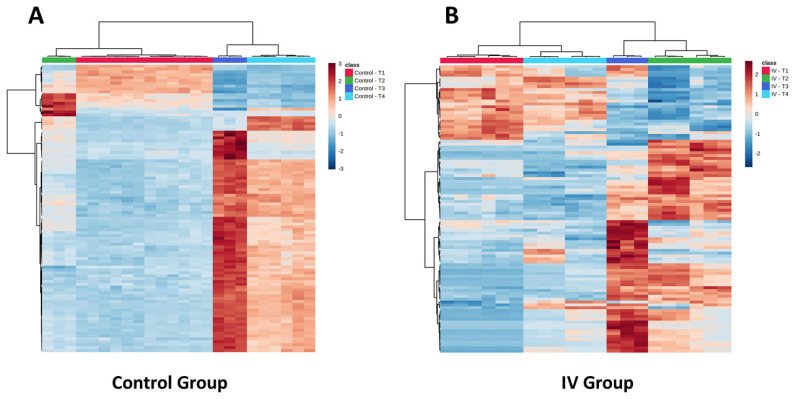
Heatmaps representing control and IV groups (positive ESI) for the top 100 discriminating features. The four time points (each urine pool in triplicate) are represented as pre-dose (T1, red) and 0–3 h (T2, green), 3–8 h (T3, dark blue), and 8–24 h (T4, light blue) post-dose. Euclidean distance and Ward clustering were applied in both cases. In the case of the control group (**A**), no urine was obtained for one of the groups at T2 and T3. Similarly, for the IV group (**B**), no sample was obtained for one group at time T3. For comparison, the pre-dose vs. post-dose urine from both the control and dosed groups are shown in (**A**) to illustrate the similarity in profiles of all animals prior to study commencement.

In order to examine the effects of exposure to gefitinib and its metabolites on the urinary excretion of endogenous metabolites, the untargeted profiling data were examined after the removal of any drug-related ions from the dataset. The inclusion criteria for endogenous metabolites to be accepted in the data for analysis were that a feature had to show a minimum of ≤30% CV variation in signal, and ideally less, in the QC samples. To obtain an indication of the technical variability each sample (from all timepoints) was analyzed in triplicate (see Figure 3). When the data from this study were analyzed, a total of 6179 features were detected in the positive ESI that met the acceptance criterion of having a CV of ≤30%. For negative ESI, the corresponding figure was 2025 ions detected with a CV ≤30%. The data for all of the QC samples are provided in Supplementary Tables S1 and S2, together with a breakdown of features showing CVs of ≤20 and ≤10%. Retention time variability was less than 0.5% for all of these features. No trends in either the signal intensity or retention time were observed over the time-course of the analysis for either the study replicates or QC samples.

As can be seen from the PCA of these urine samples in Figure 1, for the positive ESI data there was evidence for a clear time-related “trajectory” in response to gefitinib administration (see Figure S2 for the negative ESI result). Thus, the pre-dose samples clustered closely together with 0–3 and 3–8 h post-dose samples distant from them. However, the 8–24 h urine data showed evidence that the animals were returning to a more normal “pre-dose” metabolic phenotype, as these samples were mapped into a similar metabolic “space” as the pre-dose urine. It is also of interest to note that there were some cage effects, with an obvious difference between the two pools for the 0–3 h samples evident in the PCA and, to some extent, in the heatmaps shown in Figure 3.

Analysis of these data by Metaboanalyst [37] and the production of heatmaps based on changes in the top 100 features (as determined statistically by T-test and ANOVA) for the positive ESI data, demonstrated similarities between all of the mouse groups involved in the study at the pre-dose timepoint (see Figure 3A). However, as can be seen in Figure S3, for both the positive and negative ESI data, there was also evidence for diurnal variation in the vehicle group in the absence of drug administration. Diurnal variation is also apparent in Figure 3A for the control mice. However, following the administration of the drug, the urine of the gefitinib-dosed mice showed a rapid divergence from those of the undosed controls, as would be expected from the PCA. This divergence was evident in the first 0–3 h post-dose, but was also present in the data from the 3–8 h timepoint, before normalizing somewhat over the subsequent 16 h, as shown for the 8–24 h samples in Figure 3B. The examination of these data showed that the changes detected in the metabolic profiles comprised both time-related relative increases and decreases in the amounts of numerous metabolites in the urine obtained from gefitinib-dosed animals. Heatmaps illustrating the results for the top 100 changed metabolites in the control (Figure 3A) and gefitinib (Figure 3B) dosed animals provide further support for the time-course trajectory, indicated in the PCA shown in Figure 1.

Similar heatmaps for the negative ESI data are provided in Figure S4. All of these results clearly indicate that the administration of gefitinib at a subtoxic “pharmacological dose” had profound pharmacometabodynamic effects on the urinary metabotypes. As such, these changes may enable the identification of mechanistic or diagnostic endogenous metabolites that could prove useful in understanding the activity of the drug at a molecular level. We have therefore begun an attempt to characterize the various metabolites that were affected by the administration of the drug, as described below.

### 2.4. Endogenous Metabolite Identification

The use of online databases permitted the putative “annotation” of many of the metabolites detected as being changed in the urine of gefitinib-dosed mice. However, annotations are not identifications, only an indication of possibilities, and are limited by the content of the respective databases. Thus, while well-established databases (such as the HMBD) contain large numbers of compounds, many of them are not relevant to mammals as they are natural products (e.g., plant specific phytochemicals and microbial secondary metabolites) or xenobiotics, such as drugs (including gefitinib; HMDB0014462), pesticides, or industrial chemicals. In order to build meaningful models or hypotheses, these tentative annotations must be carefully curated to eliminate those that clearly lack “biological plausibility” in the context of the study. Even where those that are highlighted by such searches do provide viable candidates for further consideration, there are often a number of potential identifications for compounds with the same nominal mass/atomic composition (such as leucine and isoleucine or 1-methyl and 3-methylhistidine). For this reason, there is a need to ensure that metabolite identification for hypothesis generation, or their use as potential mechanistic markers of the biological activity of a drug or toxin, are identified to MSI Level 1 [38], if at all possible. While mass spectrometry can often reduce the search space, through atomic composition and accurate mass data combined with distinctive/characteristic fragmentation patterns, unequivocal identification may still not be possible in the absence of an authentic standard. Here, we also employed retention time (t_R_) and IM-enabled MS in the analysis as additional means of characterization.

Like many others [39,40,41], we have observed that the addition of IM provides many advantages in the characterization of metabolites in untargeted metabolic phenotyping [42,43,44]. The first benefit of IM/MS is a consequence of the fact that IM provides a second mode of separation that is orthogonal to the LC dimension. This can result in significantly improved mass spectra as co-eluting molecules, whose spectra would contribute unwanted “noise” to the MS data for the compound of interest, can be removed by IM as a result of differential mobility. This separation is the result of molecules having different, and characteristic, collision cross sections (CCS) and, in properly calibrated systems, these values can also act as an aid to identification. Thus, these CCS data can be used as further evidence of identity, either by comparison of the experimentally derived value with an authentic standard or, where these are not available, from a calculated value [44]. Even where the CCS value obtained does not confirm identification, it may help to reduce the metabolic “search space” by eliminating obviously incorrect structures.

With that being said, irrespective of the methods used for identification, the large number of candidate metabolites detected here still makes this a daunting task that we have not yet completed. However, to illustrate the potential of the approach, some examples that show how this process might aid in the investigation of the pharmacometabodynamic effects of gefitinib are provided in Table 1, and their mass spectra are in the supplementary data (Figures S5–S13).

**Figure 4:**
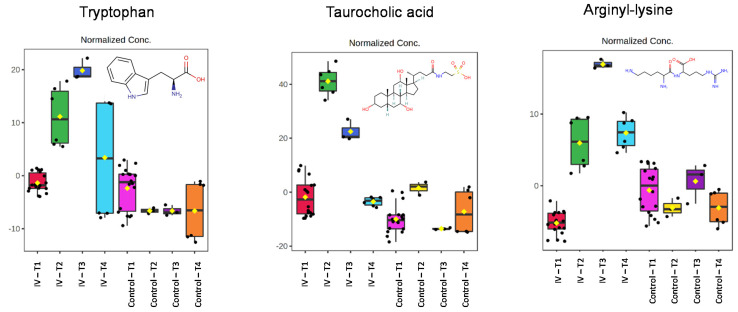
Pharmacometabodynamic variation in the urinary excretion of the endogenous metabolites of tryptophan, taurocholic acid, and arginyl-glycine (structures inset to Figure). The data for these metabolites were normalized using all features. The four time points are represented as pre-dose (T1, pink) and 0–3 h (T2, yellow), 3–8 h (T3, dark purple) and 8–24 h (T4, orange) post-dose for the control mice, and pre-dose (T1, red) and 0–3 h (T2, green), 3–8 h (T3, dark blue), and 8–24 h (T4, light blue) post-dose for gefitinib-dosed animals.

**Figure 5:**
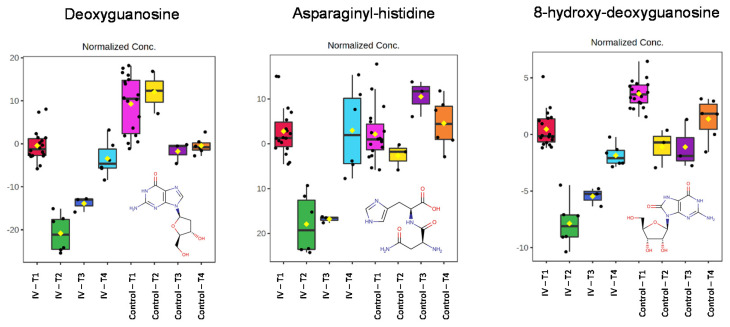
Pharmacometabodynamic variation in the urinary excretion of the endogenous metabolites deoxyguanosine, asparaginyl-histidine, and 8-hydroxydeoxyguanosine (structures inset to Figure). The data for these metabolites were normalized using all features The four time points are represented as pre-dose (T1, pink) and 0–3 h (T2, yellow), 3–8 h (T3, dark purple) and 8–24 h (T4, orange) post-dose for the control mice, and pre-dose (T1, red) and 0–3 h (T2, green), 3–8 h (T3, dark blue), and 8–24 h (T4, light blue) post-dose for gefitinib-dosed animals.

Based on the data illustrated in Figure 3, six examples were chosen to illustrate the behavior of the many endogenous metabolites seen to change as a result of exposure to gefitinib. Thus, three of the chosen metabolites that showed a relative increase in urinary concentration, together with a further three examples that, in contrast, decreased in amount, are illustrated in Figure 4 and Figure 5, respectively. These metabolites were chosen based on their profiles as being representative of the types of change being seen, and reasonable confidence in their identification to MSI 1. Clearly, in the absence of flux experiments, it is not obvious whether such changes, in these or the other metabolites showing similar excretion profiles, are the result of increased biosynthesis or decreased utilization/degradation for those apparently “upregulated” or the reverse for “downregulated” compounds.

The urinary excretion profiles of the metabolites identified as tryptophan and taurocholic acid and the dipeptide lysyl-arginine, selected to exemplify those showing a relative increase in amount compared with the control animals, are illustrated in Figure 4. These metabolites were identified in the case of tryptophan based on the characteristic mass spectrum of the protonated form, as well as the fit of the measured CCS value, to that obtained from library data/calculated data and t_R_. The mass spectra of both the [M + H] of tryptophan and its acetonitrile adduct are provided in Figures S5 and S6. Similar comments apply to taurocholic acid, with characteristic fragmentation showing the loss of taurine, etc., (see Figure S7) and a good fit for the CCS value and t_R_ (Table 1). The third example is for the dipeptide arginyl-lysine, where confident MS-characterization was relatively simple from the fragmentation pattern obtained by MS (see Figures S8 and S9).

While it would be easy to over-interpret such urinary excretion data, it would appear that the onset of the effects of gefitinib administration on the excretion of taurocholic acid were fairly transient compared with the other two metabolites. Thus, the relative increase in the amounts of taurocholic acid in the urine was rapid, peaking in the 0–3 h sample, and declining rapidly thereafter. In the cases of both tryptophan and lysyl-arginine, while they were also elevated in the 0–3 h post-dose samples, the peak amounts of these metabolites were seen in the 3–8 h post-dose urine collection.

In the case of compounds showing a relative decrease in amount compared with the controls, two metabolites of the purine nucleoside guanosine (guanine linked via a β-N9-glycosidic bond to ribose) were identified. One of these was deoxyguanosine and the other 8-hydroxydeoxyguanosine. Both were identified on the basis of the characteristic mass spectrometric fragmentation data supported by their experimentally determined CCS values, comparing well to library/calculated values (Table 1 and Figures S10–12, respectively**)**. As seen with the “upregulated” compounds, a number of dipeptides were also found to be “downregulated”. As an example, asparaginyl-histidine was easily characterized based on its fragmentation data (Figure S13). The excretion profiles of all three metabolites showed a rapid initial decline, with the lowest concentrations seen in the 0–3 h post-dose urine.

While we are confident that the identifications of the metabolites highlighted above are sound, there are clearly many questions remaining. In part, this requires the identification of many of the still only partially characterized compounds detected as changing in the urine of gefitinib-dosed animals. We intend that these remaining metabolites will be identified in future studies, but, in order to avoid developing spurious and highly speculative theories about mode of action, etc., we will only base hypotheses on compounds for which we have confident identifications. Hopefully, as more identities are confirmed it will be possible to obtain a more comprehensive picture of the nature of the biochemical response of the mouse to EGFR inhibition by gefitinib, and from that obtain a greater understanding of both the drug and biological system.

## 3. Discussion

There are some obvious limitations to the present study, the main one being that it was not specifically designed as a metabolomic investigation. The original design was to evaluate the use of state of the art UHPLC/IM/MS in defining the DMPK properties of gefitinib, using small samples and the minimum number of animals [14]. Such small scale in vivo DMPK studies are generally undertaken in rodents in the later phases of drug discovery (as a prelude to candidate selection), or early in drug development. However, in an effort to maximize the data recovery from this study, in line with the well accepted replace, reduce, and refine (3Rs) initiatives in current animal research, we explored the possibilities provided for using the remaining samples to obtain further biochemical information via metabolic phenotyping.

In addition, as noted above, the urine samples analyzed here were obtained from mice housed in groups (five animals/metabowl, see experimental), and thus represent pooled collections from each of these groups. Clearly, in an ideal situation, samples of urine would, like the blood samples, have been collected from individual animals. However, practical and ethical considerations made this impractical in the context of the overall study design.

Nevertheless, if considered as a preliminary, rather than definitive study, the results obtained show considerable promise as a means of extracting information from samples that, having served their main purpose, might otherwise have been discarded. Compared with the undosed controls, the gefitinib-dosed mice exhibited changes in the urinary content of a range of metabolites. These were maximal at a time when the circulating concentrations of gefitinib were high, normalizing with time as the drug and related metabolites were eliminated from the plasma. By 24 h post-dose, when the animals were no longer exposed to gefitinib or its metabolites in the circulation, the amounts of these endogenous the metabolites in urine were close to those of the controls (Figure 4 and Figure 5).

Changes such as these, that appear to be clearly associated with the presence of a drug, reversing in its absence, are clearly indicative of a “pharmacological” response. In parallel with pharmacodynamics, we would suggest that they represent a pharmaco-metabodynamic effect (here defined as “the dynamic, time-related, and reversible changes in metabolic phenotypes resulting from the pharmacological effects of a drug (or other bioactive substance) on the metabolome”). To substantiate this conjecture, further experiments using a study specifically designed to prove the linkage of drug and metabolite response are indicated.

However, notwithstanding the need for further evidence, it is possible to speculate that the rapid increase in the relative amounts of the three endogenous metabolites used as examples above (tryptophan, taurocholic acid, and arginyl-glycine) that were seen to be increased in amount in the urine (Figure 4) reflect the early effects of gefitinib on, e.g., the liver. This might have been the result of, e.g., a reduction in bile flow, with taurocholic acid then entering the circulation and being excreted via the urine. As the circulating concentrations of gefitinib rapidly decreased, its effects on the liver would have reduced and, as biliary homeostasis was restored, the urinary concentrations of taurocholic acid would normalize. However, without actual measurements of bile concentrations, and a targeted, and quantitative assay for these compounds in both circulation and urine, this sort of “explanation” is merely speculation. With tryptophan, it can be conjectured that the more gradual increase in its concentrations in the urine, peaking well after the maximum plasma concentrations of gefitinib, were the result of the decreased utilization/degradation of this essential amino acid or increased protein degradation. The function of the dipeptide, which followed a similar time course to tryptophan, is more obscure and, at this time, we do not feel able to comment on it further.

The opposite behavior was seen with deoxyguanosine, asparaginyl-histidine, and 8-hydroxydeoxyguanosine, which declined in amount in the 0–3 h urine samples, when gefitinib concentrations in the circulation were high, but rapidly returned to more normal quantities in the subsequent 3–8 h samples and to control values by 8–24 h post-dose (Figure 5). This profile closely follows the plasma pharmacokinetics of the drug and its metabolites again, suggesting a clear and direct pharmacometabodynamic effect. The effects on deoxyguanosine and 8-hydroxydeoxyguanosine are intriguing, suggesting that gefitinib has some downstream effects on DNA (8-hydroxydeoxyguanosine has been used as a biomarker of oxidative DNA damage [45]) that might bear further investigation. While this is once more clearly speculation at the moment, it provides fertile ground for hypothesis generation that could be investigated further (either using bespoke in vitro/in vivo investigations or by further targeted analyses of these samples).

Based on the review by Poliakova et al. [46] of the known biochemical effects of TKI inhibitors on a broad spectrum of pathways, including the tricarboxylic acid cycle, glycolysis, lipid and amino acid metabolism, the fact that gefitinib has effects on the metabolome should not be considered surprising. To date, investigations of the metabolome-wide effects of TKI inhibitors have been somewhat limited in scope. However, one such investigation has been undertaken on the TKI inhibitors sunitinib and erlotinib. Both drugs were administered to mice for 2 weeks, at which point the serum, heart, skeletal muscle, and liver, but not urine, were taken for analysis. This study provided evidence for metabolic changes when profiled using un-targeted GC-MS [47]. In the case of sunitinib, a cardiotoxin, significant decreases in O-phosphocolamine, 6-hydroxynicotinic acid, docosahexaenoic acid (DHA), arachidonic acid (AA), and eicosapentaenoic acid (EPA) were seen in the heart, and DHA was also lower in the skeletal muscle. For serum and liver, raised amounts of ethanolamine and cholesterol, respectively, were observed together with decreases in liver dehydroalanine, adenosine, and docosahexaenoic acid. For erlotinib, increased serum threonic acid, a C14 hydrocarbon, was noted, with decreased liver homoserine and ornithine. Erlotinib administration resulted in raised spermidine in the heart.

As Poliakova et al. [46] say, in the concluding sentence of their review “…although the current knowledge on TKIs impact on cellular metabolism is continuously expanding, the detailed molecular mechanisms underlying many of the observations described within this review remain largely unknown” concluding that “… further biological investigations are warranted to understand the metabolic on- and off-target effects related to TKIs treatment”.

The application of UPLC/IM/MS to the analysis of urine, as well as other sample types, combined with the linkage to drug pharmacokinetics, as demonstrated here, offers a practical approach to the study of the global changes in the metabolite profiles resulting from exposure to TKIs, and should be equally applicable to other drug classes.

## 4. Materials and Methods

### 4.1. Chemicals and Reagents

LC/MS grade water, acetonitrile (ACN), and formic acid (FA) were all acquired from Fluka (Loughborough, UK). Authentic standards and sodium formate (MS calibrant) were sourced from Sigma Aldrich. Instrument calibration used the “Waters Major Mix IMS/ToF Calibration Kit for IMS” (Waters Corp., Milford, USA). Leucine Enkephalin (Sigma, Dorset, UK) was used as the lockmass calibrant MS.

### 4.2. Study Conduct

A detailed description of the study is provided in [14]. Briefly, gefitinib or vehicle were dosed intravenously (IV) via the tail vein, at 10 mg/kg (Evotec SAS, Tolouse, France) to male C57Bl/6JRj mice (n = 10/group, 9 weeks of age, 20.3–26.5 g). The dose solution (1 mg/mL) was a clear solution in hydroxypropyl-β-cyclodextrin (HPBCD) at pH 4.0 in a 50 mM acetate buffer (10:90 *w/v*) and was administered at a rate of 10mL/kg. A full management review of the study was performed to ensure that the design conformed to both National and EU guidelines prior to study commencement. Following drug administration, the mice were housed by dose group (two groups of five for both vehicle and gefitinib-dosed animals) in “metabowls” to facilitate in the collection of urine. Urine was obtained pre-dose (overnight collection) and for 0–3, 3–8, and 8–24 h post-administration. After collection, urine samples were stored at −80 °C until transfer (on solid carbon dioxide) to Waters Corp., (Wilmslow, UK) and were stored at −80 °C until analysis, as described below.

### 4.3. Metabolite Profiling

The metabolite profiling for both endogenous and gefitinib and drug-related metabolites aliquots (20 µL) of each of the urine samples were mixed, in 1.5 mL centrifuge tubes, with an equal volume of LCMS grade water. To this sample, 350 µL of LCMS grade ACN was added. Following vortex mixing, samples were kept at 2–8 °C for 10 min before centrifugation (13,000 rcf, 10 min). An aliquot of 150 µL of clear supernatant was then taken from each sample and was mixed with an equal volume of LC/MS grade water. From each sample, 20 µL were taken and mixed to provide a pooled sample for use as a quality control (QC) sample [48,49,50]. While the study samples were randomized for analysis (in triplicate), the QC samples were run at regular intervals (every five samples) throughout the course of the analysis. Prior to analysis, five of the QC samples were run in order to condition the column and to ensure retention time stability during the analysis.

### 4.4. Reversed-Phase LC/IM/MS

The sample analysis was performed using an I-Class ACQUITY PREMIER UPLC configured with a binary solvent manager, sample manager, and column oven (Waters Corp., Milford, MA, USA), with separation on a 2.1 × 100 mm, 1.8 µm HSS T3 ACQUITY PREMIER column (Waters Corp., Milford, MA, USA). The elution solvents were (A) 0.1% (*v/v*) FA in water and (B) 0.1% (*v/v*) FA in ACN. RPLC/MS was carried out using a column temperature of 40 °C and a solvent flow rate of 0.5 mL/min, via a multi-linear solvent gradient beginning with 99% solvent A, which was held for 1 min, following which the proportion of solvent B was raised in a linear fashion to 15% (3 min), 50% (6 min), and 95% (9 min). At 10 min after injection, the solvent was returned to 99% A, and the column was re-equilibrated for 2 min prior to the next sample [51].

The MS data were collected using positive (ESI+) and negative (ESI-) electrospray ionization with a SYNAPT XS mass spectrometer (Waters Corp., Wilmslow, UK) in continuum mode using the acquisition mode HDMSE [52,53]. A mass (m/z) range of 50–1200 amu and a scan time of 0.1 s were employed. A low (MS1) collision energy of 4 eV was utilized to gain precursor information, while elevated energy (MS2) via a linear collision energy ramp from 19 to 45 eV was employed to provide fragment ion data. The lockspray, containing leucine enkephalin at 200 pg/µL, was infused at 20 µL/min, and was acquired every 30 s to ensure mass accuracy. A capillary voltage of 1.0 kV (ESI+), 2.0 kV (ESI-), a cone voltage of 25 V, and a source temperature set to 120 °C were used. The flow rate of the cone gas (nitrogen) was 50 L/h and the flow rate of the nebulization gas (also nitrogen) was 800 L/h. A desolvation gas temperature of 600 °C was used. In the case of the ion mobility settings, the T-wave velocity was 650 m/s and a pulse height of 40 V was employed. The drift gas employed for IM was nitrogen (180 mL/min) with calibration over the CCS range = 130–306 Å^2^ performed using the Major Mix IMS calibration kit. Calibration of the TOF over the acquisition mass range used 0.5 mM sodium formate, and the data were collected with MassLynx vs. 4.2 software (Waters Corp., Wilmslow, UK).

### 4.5. Data Analysis for Metabolite Identification

LC/MS data were aligned and normalized using Progenesis QI (Nonlinear Dynamics, Newcastle upon Tyne, UK). The data were aligned using a study pooled QC and normalized using all of the compounds. Based on the aligned runs, an aggregate file was constructed to allow for peak picking and to eliminate potential missing values. The processed dataset was further interrogated using a variety of statistical analysis tools, including EZInfo (Umetrics, Umeå, Sweden) and MetaboAnalyst [37]. Multivariate statistical analysis was conducted using unsupervised PCA to determine the group differences. Pareto scaling was used, in which each variable was centered and multiplied by 1/√S_K_, where S_K_ is the standard deviation of the variable. Hierarchical clustering and Pearson’s R coefficient were used for the correlation analysis and pattern searching. Molecular features resulting from the statistical analysis were identified based on accurate mass, isotopic fit, and matching of in-silico fragmentation spectra using a combination of compound databases, including Human Metabolite Database (HMDB vs.4.0) and ChemSpider (vs. 1.0.7075.38452) these databases were accessed between August and December 2020. The measured CCS values for statistically relevant molecular features were also compared with theoretically derived CCS values, using an in-house CCS prediction algorithm [44].

Features corresponding to endogenous metabolites, which were putatively identified by the in silico searches, were compared with authentic standards wherever possible to further confirm their identities. An in-house database of standards was used to obtain the MS (with precursor and fragmentation ion accuracy set to 5 and 10 ppm respectively), retention time (±0.5 min), and CCS (±2.5%) data.

## 5. Conclusions

Developments in modern analytical technologies provide opportunities to improve throughput and increase the efficiency of modern drug discovery. Previously, despite the limitations imposed by small samples, it has been demonstrated that it is possible, in a single in vivo study, to characterize both the DMPK properties and excretion profiles of the drug gefitinib and its metabolites in urine [14]. Here, we have shown, using untargeted metabolic phenotyping on the same urine samples, time-related changes in the urinary metabotypes for the endogenous metabolites excreted in the urine that correlate with the circulating concentrations of the drug and its metabolites. Thus, the largest changes in endogenous metabolites coincided with the highest observed plasma concentrations of gefitinib, and returned to control values as the drug concentrations fell. These pharmacometabodynamic responses have the potential to provide opportunities to examine both on and off target effects of drugs (and their metabolites). Such knowledge may enable a better understanding of the mode of drug action to be obtained at an early stage in drug discovery, and facilitate the discovery of biomarkers that can be used in the translation of animal models to patients.

## Figures and Tables

**Table 1:** Analytical data for metabolites identified in positive ESI MS as significantly contributing to the PCA.

Compound	Adduct	Experimental RT (min)	Authentic Standard RT (min)	Experimental CCS (Å)	Predicted CCS (Å)	ΔCCS Experimental vs. Predicted (%)	Authentic Standard CCS (Å)	ΔCCS Measured vs. Predicted (%)
**Tryptophan**	[M + H]	3.59	3.22	144.1	141.9	1.5	143.8	0.3
**Taurocholic acid**	[M − H]	5.77	5.94	205.9	205.9	0	207	0.5
**Arginyl-lysine**	[M + H]	5.04	n/a	166.0	173.0	3.0	n/a	-
**Arginyl-lysine**	[M − H]	5.03	n/a	167.2	172.8	3.2	n/a	-
**Deoxyguanosine**	[M + H]	0.69	0.7	155.7	154.1	1.0	153.7	1.3
**Asparaginyl-histidine**	[M + H]	2.68	n/a	157.9	156.7	0.8	n/a	-
**8-hydroxy-deoxyguanosine**	[M + H]	2.24	2.51	159.8	158.4	0.9	n/a	-

## Data Availability

All relevant data are provided in the manuscript or the Supplementary Materials.

## Supplementary Materials

The following are available online at https://www.mdpi.com/article/10.3390/metabo11060379/s1. Structure S1: Gefitinib. Figure S1: Intravenous pharmacokinetics (10 mg/kg) of gefitinib, M605211, and the O-desmethyl metabolites in the mouse. Figure S2: PCA of negative ESI data. Figure S3: PCA of both positive and negative ESI data for control animals. Figure S4: Heatmaps representing control and IV groups. Figure S5: Tryptophan fragmentation, positive ESI. Figure S6: Tryptophan acetonitrile adduct, positive ESI. Figure S7: Taurocholic acid fragmentation, negative ESI. Figure S8: Arginyl-lysine, positive ESI. Figure S9: Arginyl-lysine, negative ESI. Figure S10: Deoxyguanosine, positive ESI. Figure S11: Deoxyguanosine, acetonitrile adduct, positive ESI. Figure S12: 8-Hydroxy-deoxyguanosine, positive ESI. Figure S13: Asparaginyl-histidine, positive ESI. Table S1: Positive ion data for pooled QC data. Table S2: Negative ion data for pooled QC data.
