# Supplementary material for: The Pharmacometabodynamics of Gefitinib after Intravenous Administration to Mice: A Preliminary UPLC–IM–MS Study
Source: Metabolites. 2021 Jun 11;11(6):379. doi: 10.3390/metabo11060379 (PMC8230777; doi:10.3390/metabo11060379)
Supplement: Supplementary file 1 [file metabolites-11-00379-s001.zip › metabolites-1214410-supplementary.pdf]

The Pharmacometabodynamics of Gefitinib after Intravenous Administration to Mice: A Preliminary UPLC–IM–MS Study

Billy Molloy[1], Lauren Mullin[1], Adam King[1], Lee A. Gethings[1], Robert S. Plumb[2], Ian D Wilson[3*]

[1] Waters Corporation, Stamford Rd, Wilmslow, SK9 4AX, UK

[2]Waters Corporation, Milford, MA, 01757, USA.

[3]Division of Systems Medicine, Department of Metabolism Department of Metabolism, Digestion and Reproduction, Imperial College London, UK.

Structure S1: Gefitinib

Figure S1: Intravenous pharmacokinetics (10 mg/kg) of gefitinib, M605211 and the O-desmethyl metabolites in the mouse

Figure S2 :PCA of negative ESI data for animals dosed with gefitinib by the IV route at 10 mg/kg. The four time points are represented as pre-dose (T1), 0-3 h (T2), 3-8 h (T3) and 8-24 h (T4).

Figure S3: PCA of both positive and negative ESI data for control animals. The four time points are represented as pre-dose (T1), 0-3 h (T2), 3-8 h (T3) and 8-24 h (T4)

Figure S4: Heatmaps representing control and IV groups (for negative ESI). The four time points are represented as pre-dose (T1), 0-3 h (T2), 3-8 h (T3) and 8-24 h (T4). Euclidean distance and Ward clustering were applied in both cases. In the case of the control group no urine was obtained for one group at each of T2, and T3, similarly for the IV group no sample was obtained for one group at time T3. For comparison all the predose urines from both dosed and control groups are shown in A.

Figure S5: Tryptophan fragmentation, positive ESI.

Figure S6: Tryptophan acetonitrile adduct, positive ESI

Figure S7: Taurocholic acid fragmentation, negative ESI.

Figure S8: Arginyl-lysine, positive ESI.

Figure S9: Arginyl-lysine, negative ESI.

Figure S10: Deoxyguanosine, positive ESI.

Figure S11: Deoxyguanosine, acetonitrile adduct, positive ESI

Figure S12: 8-Hydroxydeoxyguanosine, positive ESI

Figure S13: Asparaginyl-histidine, positive ESI.

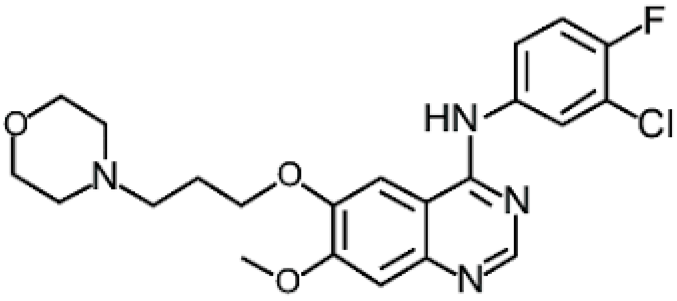

Structure S1: Gefitinib

Dark Blue = gefitinib

Red = O desmethyl

Light Blue = M605211

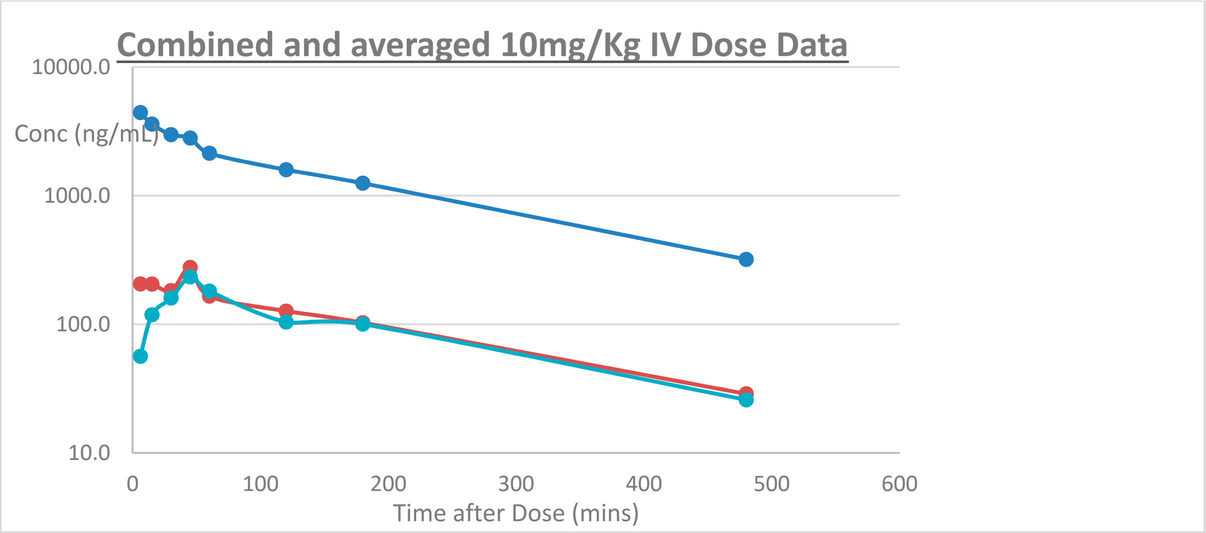

PCA – IV groups only (negative ion)

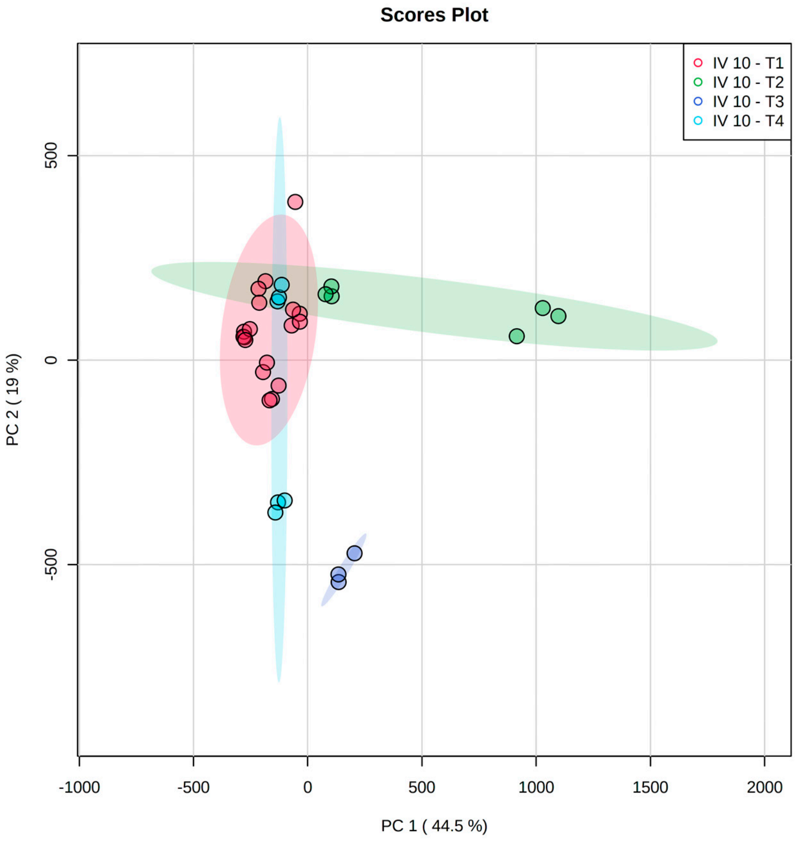

Figure S2: PCA of negative ESI data for animals dosed with gefitinib by the IV route at 10 mg/kg. The four time points are represented as pre-dose (T1), 0-3 h (T2), 3-8 h (T3) and 8-24 h (T4).

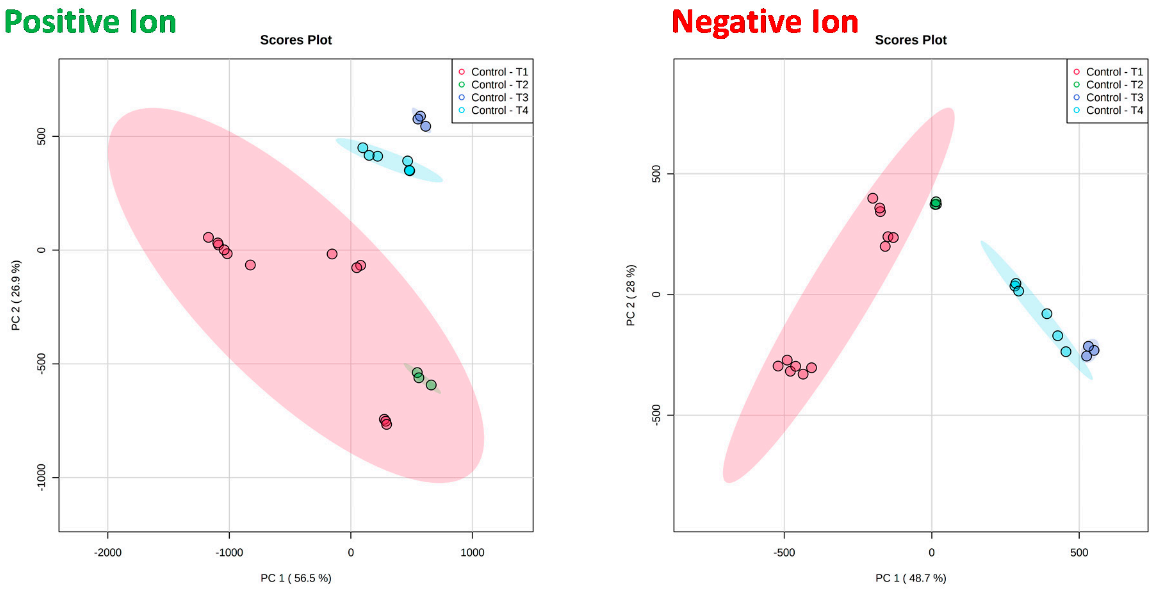

Figure S3: PCA of both positive and negative ESI data for control animals. The four time points are represented as pre-dose (T1), 0-3 h (T2), 3-8 h (T3) and 8-24 h (T4)

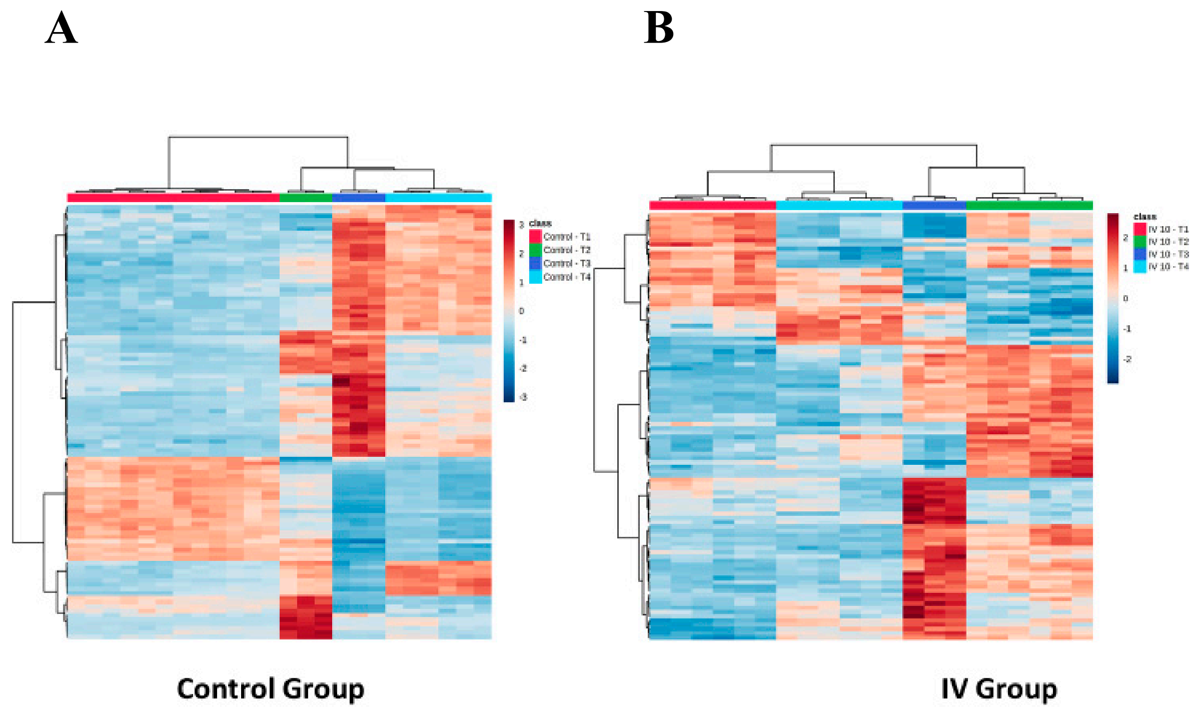

Figure S4: Heatmaps representing control and IV groups (for negative ESI). The four time points are represented as pre-dose (T1, red), 0-3 h (T2, green), 3-8 h (T3, dark blue) and 8-24 h (T4, light blue). Euclidean distance and Ward clustering were applied in both cases. In the case of the control group no urine was obtained for one group at each of T2, and T3, similarly for the IV group no sample was obtained for one group at time T3. For comparison all the predose urines from both dosed and control groups are shown in A.

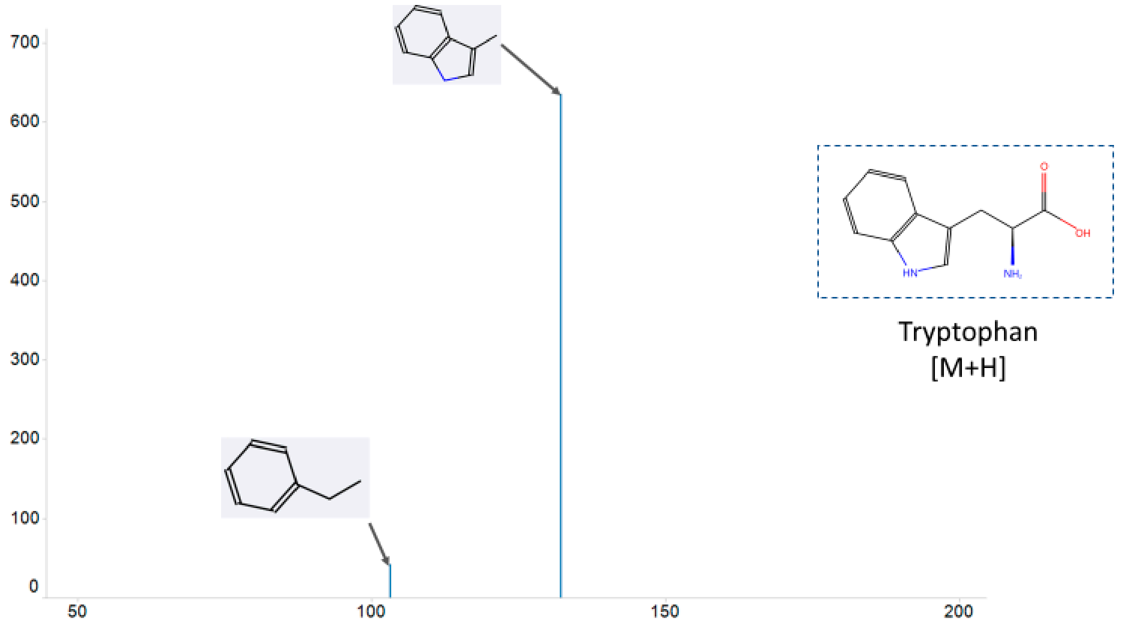

Figure S5: Tryptophan fragmentation, positive ESI

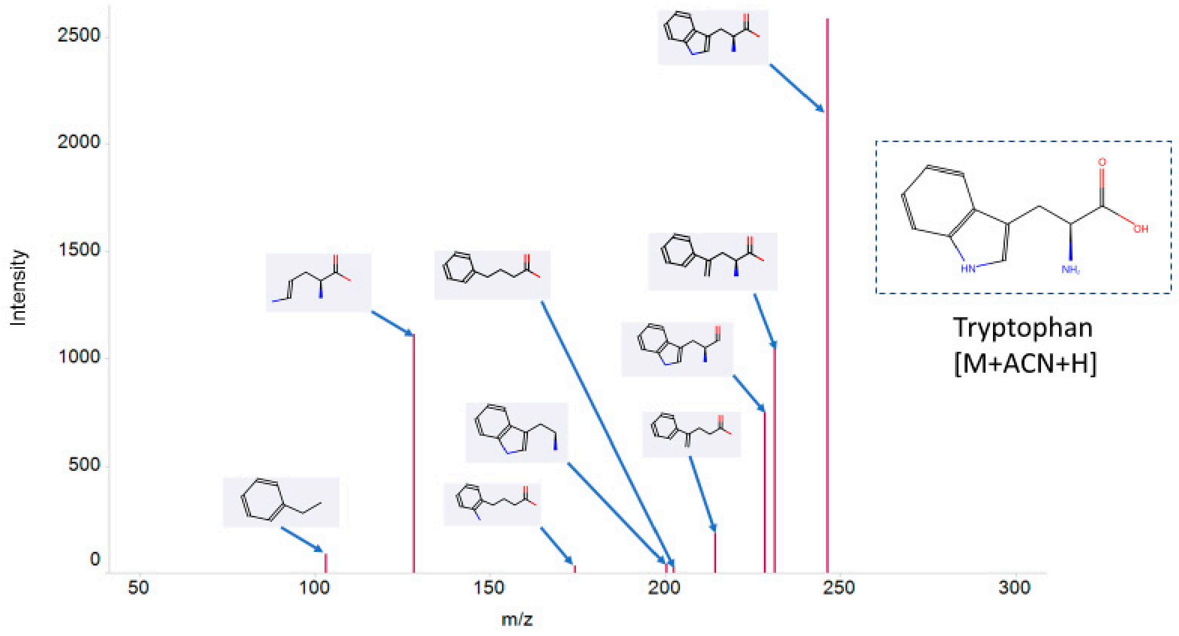

Figure S6: Tryptophan acetonitrile adduct. Positive ESI

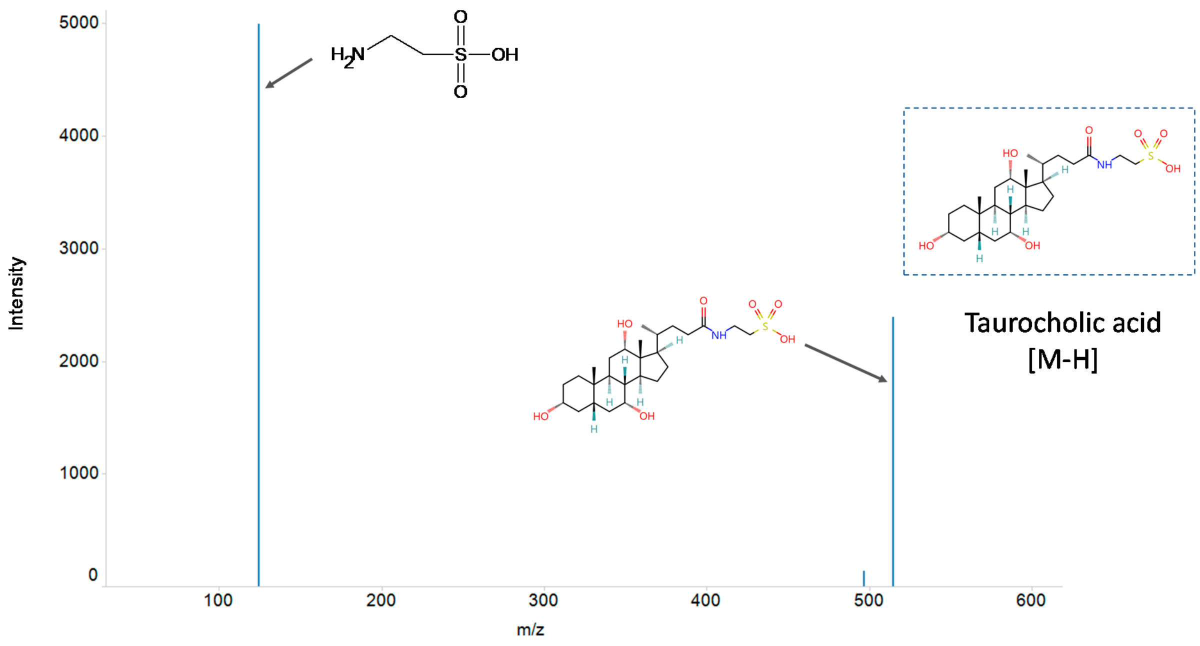

Figure S7: Taurocholic acid fragmentation, negative ESI.

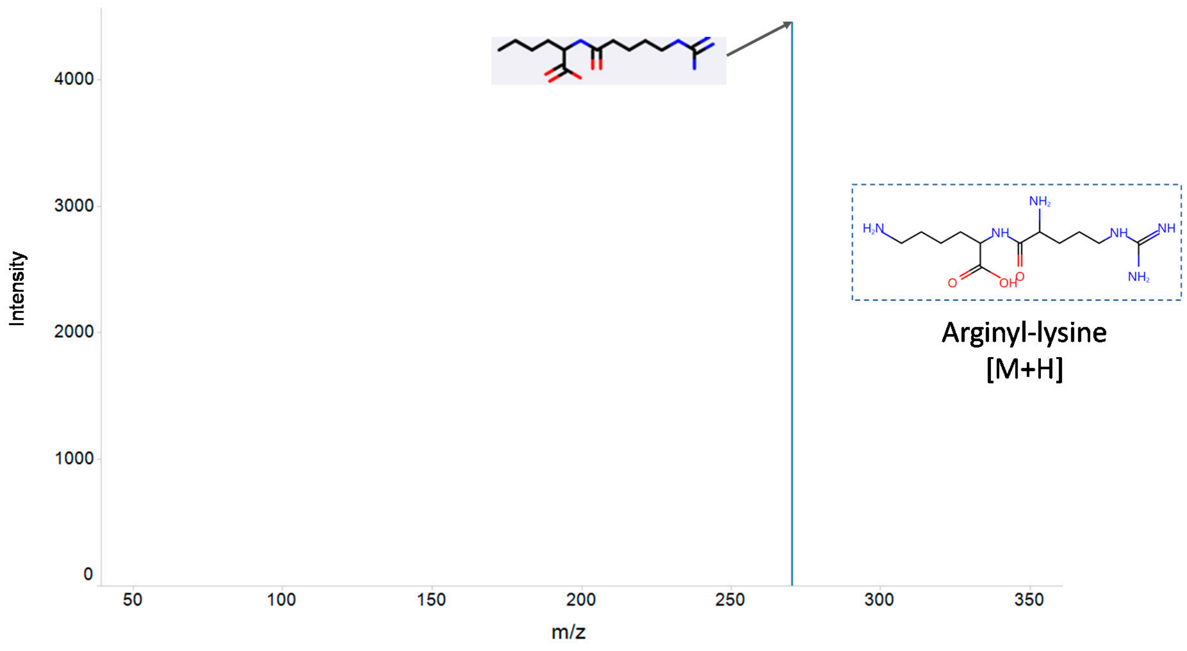

Figure S8: Arginyl-lysine, positive ESI.

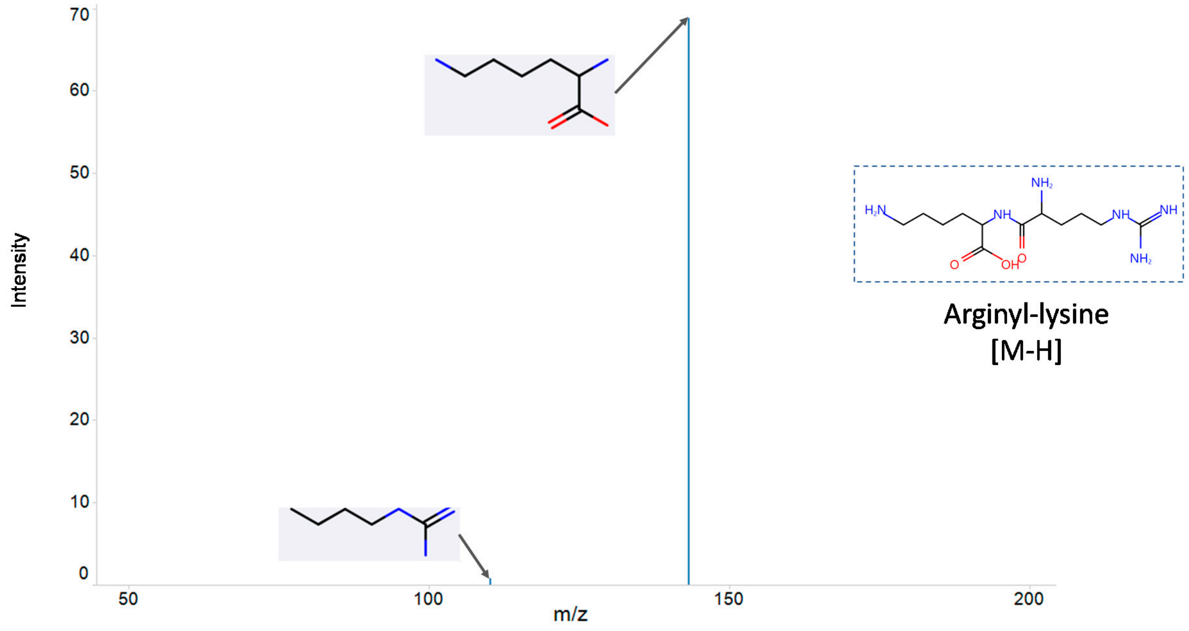

Figure S9: Arginyl-lysine, negative ESI

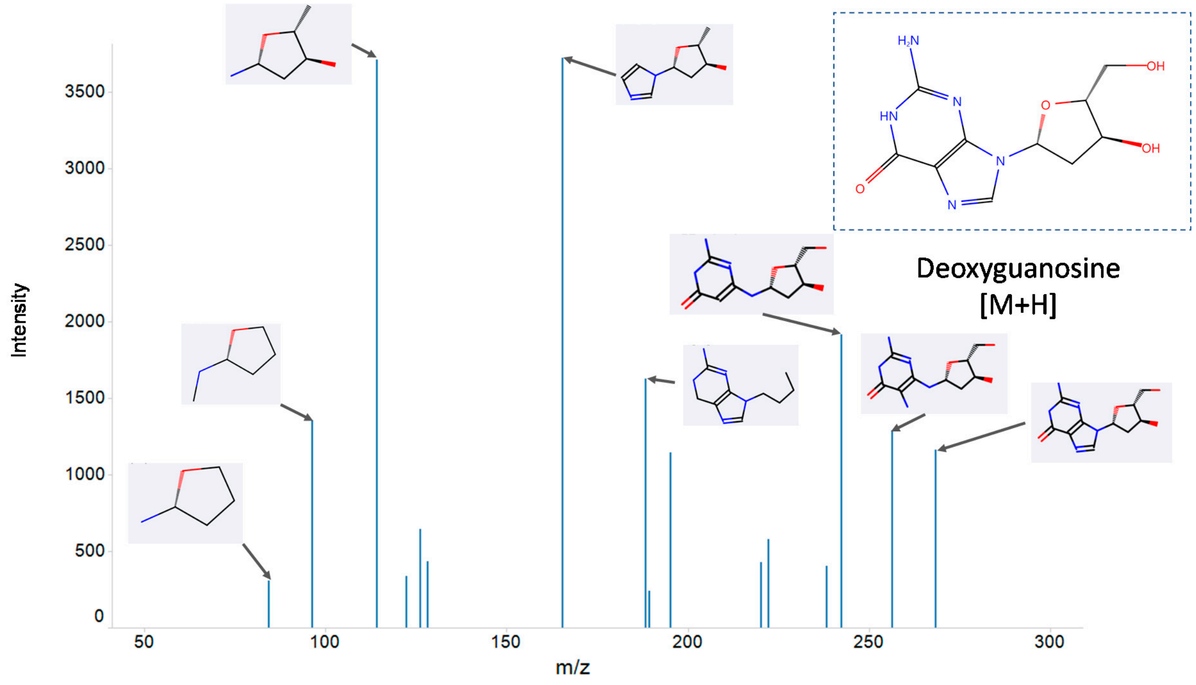

Figure S10: Deoxyguanosine, positive ESI

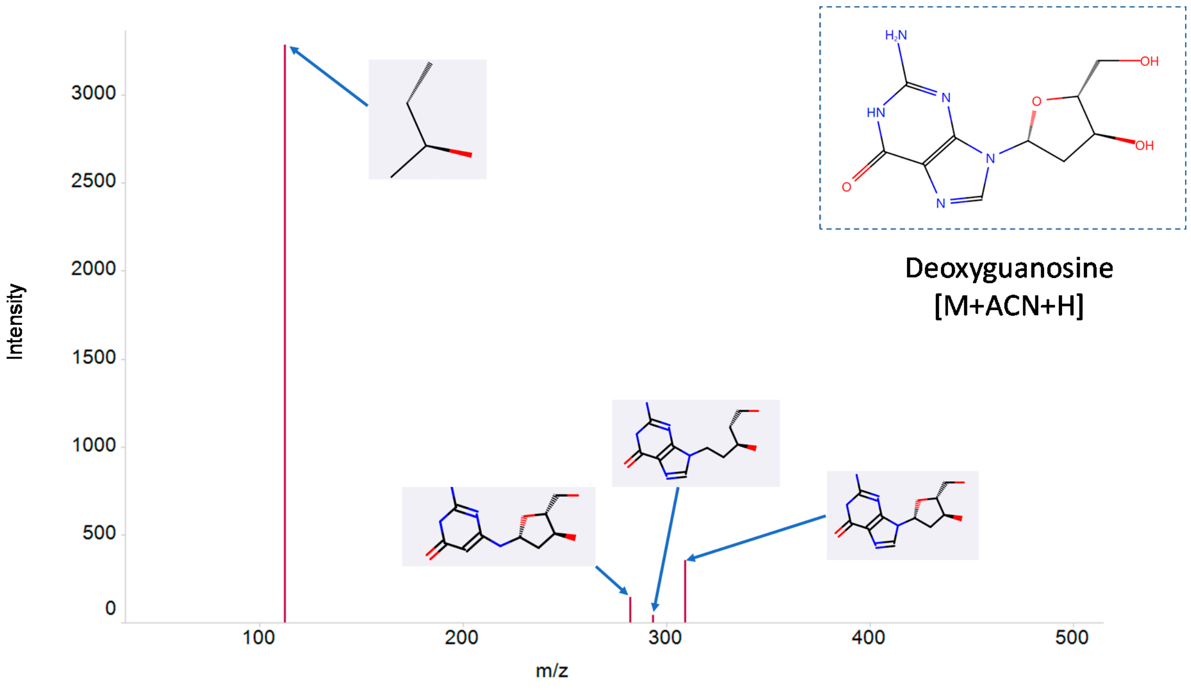

Figure S11: Deoxyguanosine, acetonitrile adduct positive ESI.

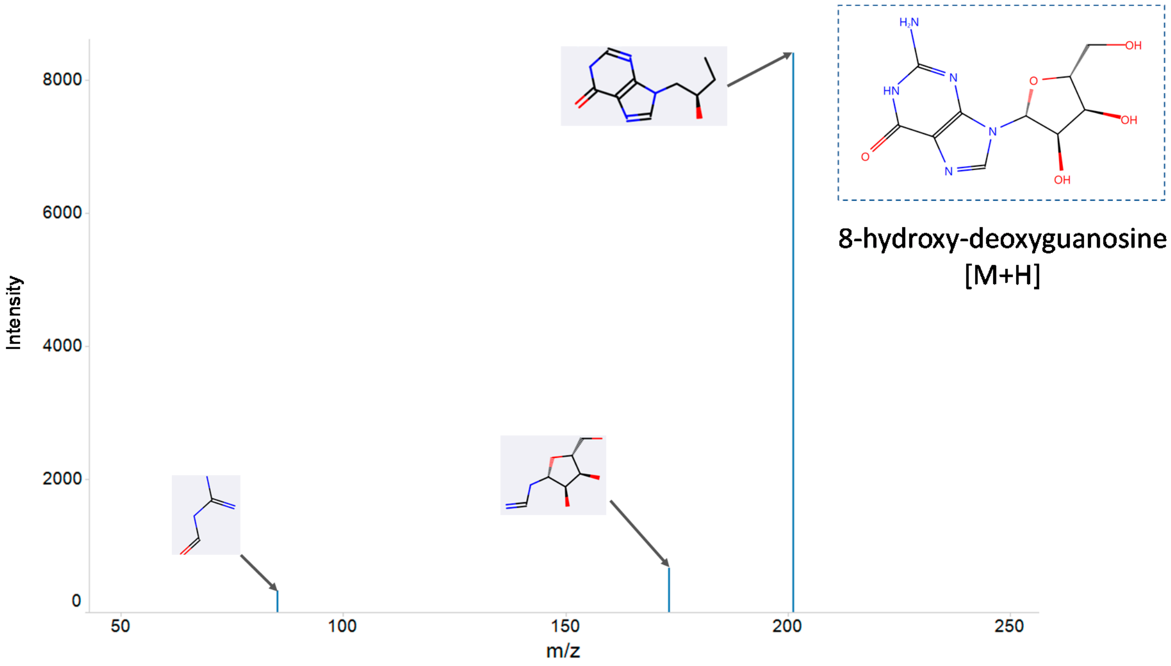

Figure S12: 8-Hydroxydeoxyguanosine, positive ESI

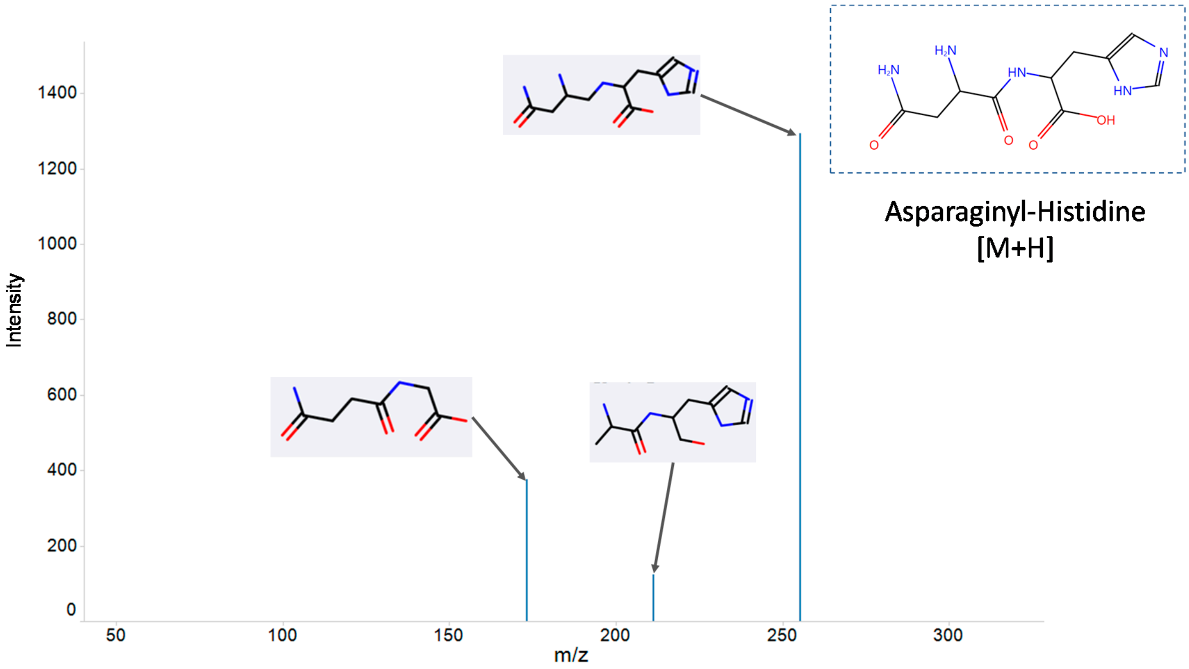

Figure S13: Asparaginyl-histidine, positive ESI.
